# Supplementary material for: Revealing the hidden carbon in forested wetland soils
Source: Nat Commun. 2024 Jan 25;15:726. doi: 10.1038/s41467-024-44888-x (PMC10810814; doi:10.1038/s41467-024-44888-x)
Supplement: Supplementary file 1 — Supplementary Information [file 41467_2024_44888_MOESM1_ESM.pdf]

# Revealing the hidden carbon in forested wetland soils

## Supplementary Material


### Author List

Anthony J Stewart*[1], email ajs0428@uw.edu
Meghan Halabisky[1]
Chad Babcock[2]
David E. Butman[1]
David V. D'Amore[3]
L. Monika Moskal[1]

### Affiliations

[1]School of Environmental and Forest Sciences, University of Washington, Seattle, Washington, United States
[2]Department of Forest Resources, University of Minnesota, St Paul, Minnesota, United States
[3]Pacific Northwest Research Station, U.S. Department of Agriculture Forest Service, Juneau, Alaska, United States


### Methods: The Wetland Intrinsic Potential Tool

We rely on the Wetland Intrinsic Potential (WIP) tool to generate the continuous wetland-to-upland gradient across the Hoh River Watershed (HRW). The details of building, training, and executing the model are described in Halabisky et al. (2023)[1] and we refer readers to there for further and more detailed information. However, to provide more insight into our use of the tool, we describe the WIP model briefly here.

The WIP model is a geospatial wetland mapping model using multiple metrics corresponding to topography, hydrology, soil characteristics, and vegetation in a random forest algorithm (Supplementary Fig. 1). There is an emphasis on topographic metrics within the WIP due to the thick forest canopy in the HRW that obscures potential wetlands. Additionally these topography metrics are calculated at multiple scales, enabling cross-scale variables to inform the wetland prediction model whereas many other studies only utilize single scale variables[2]. The total number of variables used in the model was 19.

Training data for the model were initially developed using first with 1000/2000 wetland/non-wetland locations based on maps from the National Wetland Inventory (NWI). Using the random forest with the 19 predictors, this generated a preliminary model of wetland probability across the HRW with pixel values on a scale from 0.0-1.0, with 1.0 being the maximum probability of being a wetland which we translate to a percentage (0%-100% wetland) from this point onward. We note that presence and absence based on the NWI can contain many misclassified points due to the assumption that there were many missing wetlands. Therefore, another training dataset was developed from a stratification of the NWI-derived preliminary model and sampling points in bins of the probability range (0%-25%, 25%-50%, 50%-75%, and 75%-100%). This provided a way to identify areas where there is high model uncertainty (i.e., probability near 50%). A total of 598 sample points were equally split among bins and validated using site visits and aerial photo interpretation (two points were removed due to unclear wetland/non-wetland boundary). The final random forest algorithm was run with these 600 points and the 19 predictors. A validation dataset of 100 wetland points and 200 upland points were sampled from the output of the random forest model mapped onto the HRW and were also validated with site visits and aerial photos. With wetland and non-wetland defined as above and below 50%, respectively overall accuracy of the WIP according to validation data was 91.77%.

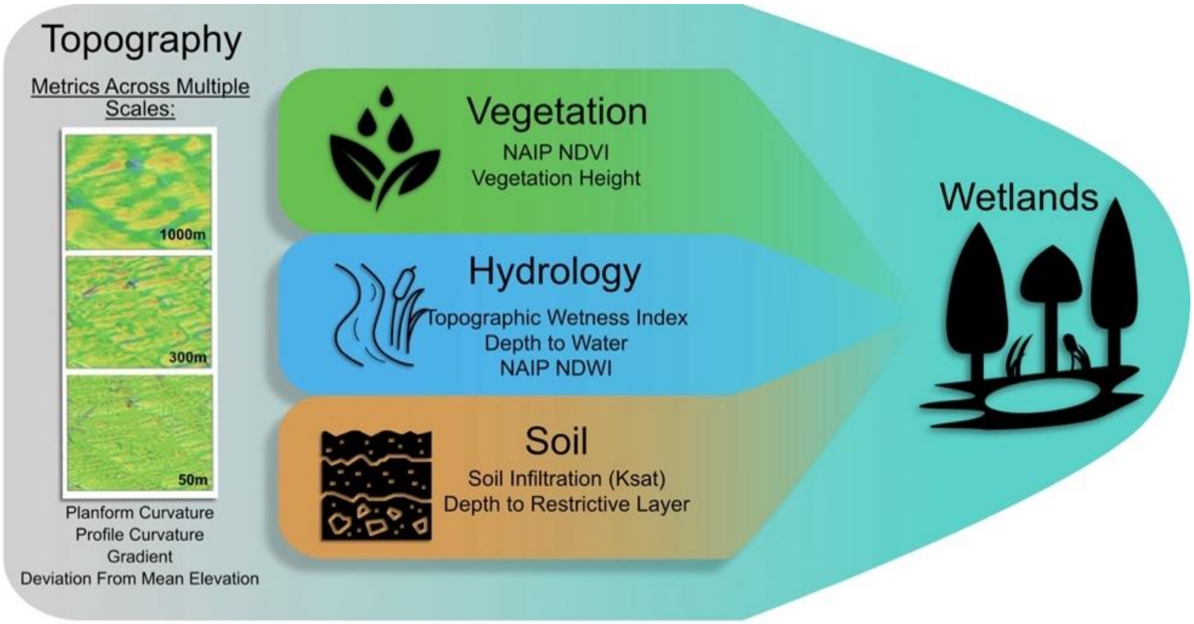


Supplementary Figure 1. **Conceptual model of the Wetland Intrinsic Potential (WIP) Tool** The National Agriculture Imagery Program (NAIP) provided fine resolution imagery for normalized difference vegetation index (NDVI) and normalized difference water index (NDWI). The conceptual model is unaltered and was originally published as Figure 2 in Halabisky et al. (2023)[1] under the Creative Commons Attribution 4.0 License (https://creativecommons.org/licenses/by/4.0/).

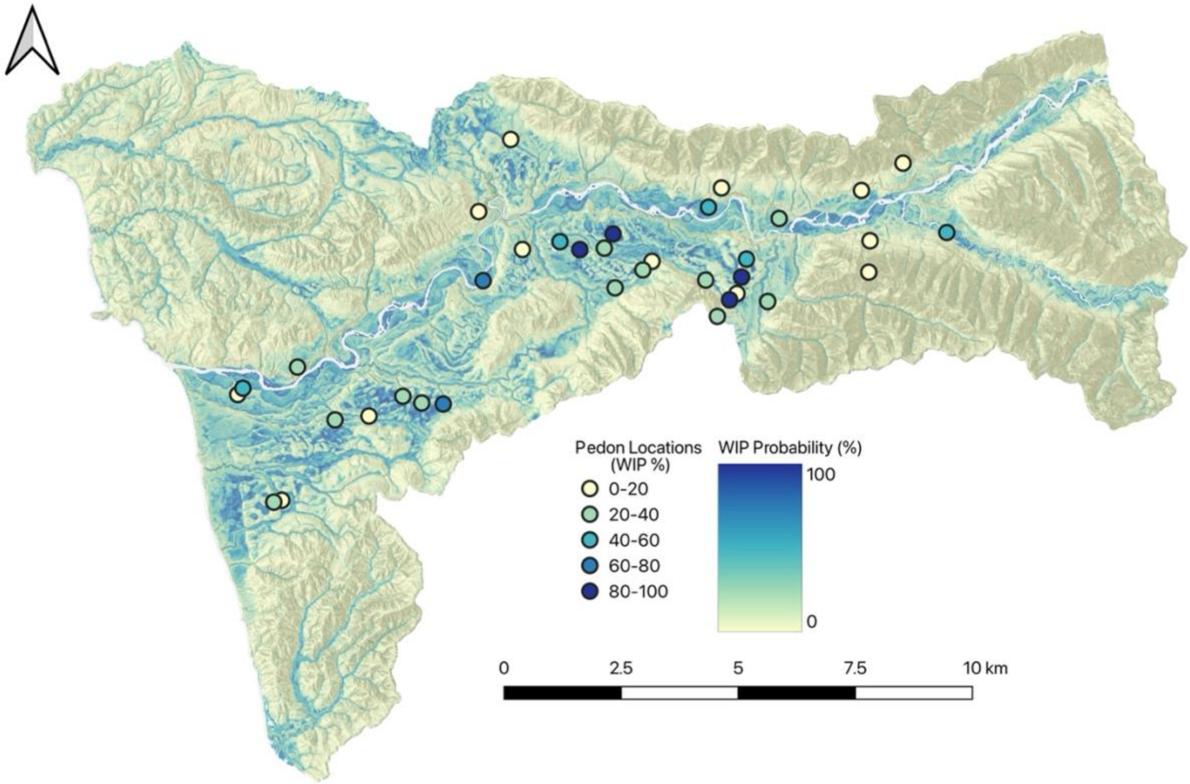


Supplementary Figure 2. **Locations for pedon soil sample collections on a map of the wetland probability from the Wetland Intrinsic Potential (WIP) tool.** WIP probability values are mapped continuously across the HRW according to the shading for probability (%). A corresponding WIP value was extracted for each location and shown in the icon. We added a semi-transparent hill shade layer to highlight terrain.

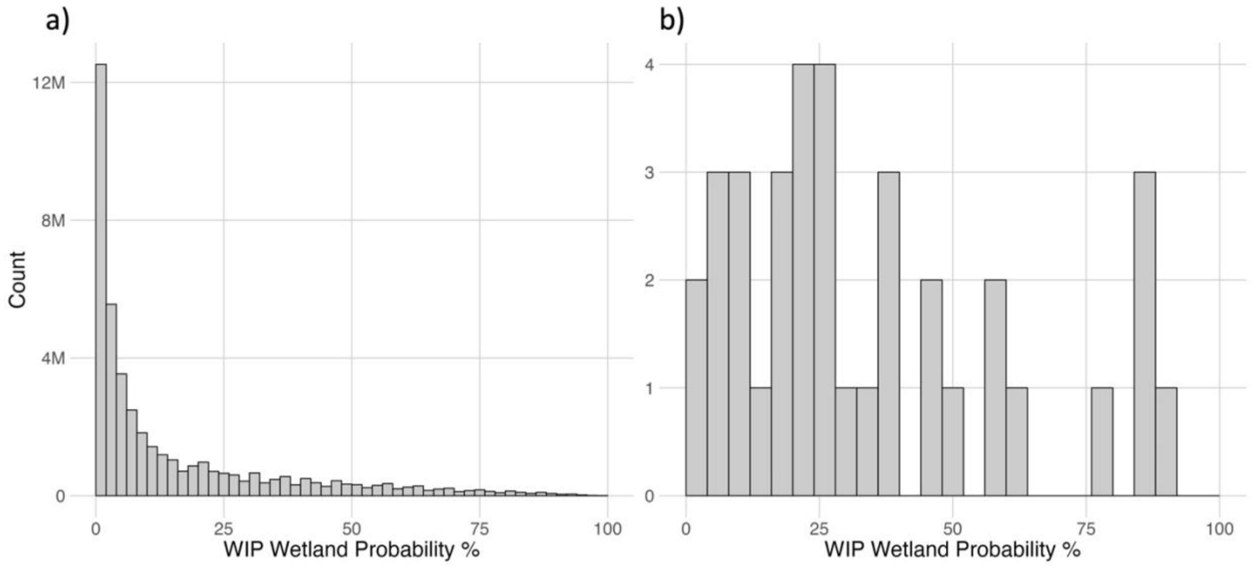


Supplementary Figure 3. **Histograms of the Wetland Intrinsic Potential (WIP) tool probability values from maps and pedons** a) Histogram distribution of the WIP map shown with the mapped area in Supplementary Figure 2; b) Histogram distribution of pedon locations also shown with points in Supplementary Figure 2.

Supplementary Table 1. **Confidence intervals of the transformed and untransformed models of the 1 m soil organic carbon (SOC) stock.** The random effect refers to the surficial geology. Sigma refers to the overall model error. The Wetland Intrinsic Potential (WIP) is the fixed effect.

| | **Square Root Transformed** | | | **Non-Transformed** | | |
|---|---|---|---|---|---|---|
| **Parameter** | **2.5%** | **Mean** | **97.5%** | **2.5%** | **Mean** | **97.5%** |
| Random Effect Standard Deviation | 2.77 | 4.65 | 6.01 | 46.7 | 108 | 141 |
| Sigma | 2.56 | 3.39 | 4.23 | 75.9 | 102 | 126 |
| Model Intercept | 6.71 | 8.42 | 10.7 | -0.39 | 48.6 | 123 |
| WIP | 6.28 | 11.0 | 15.5 | 241 | 391 | 516 |

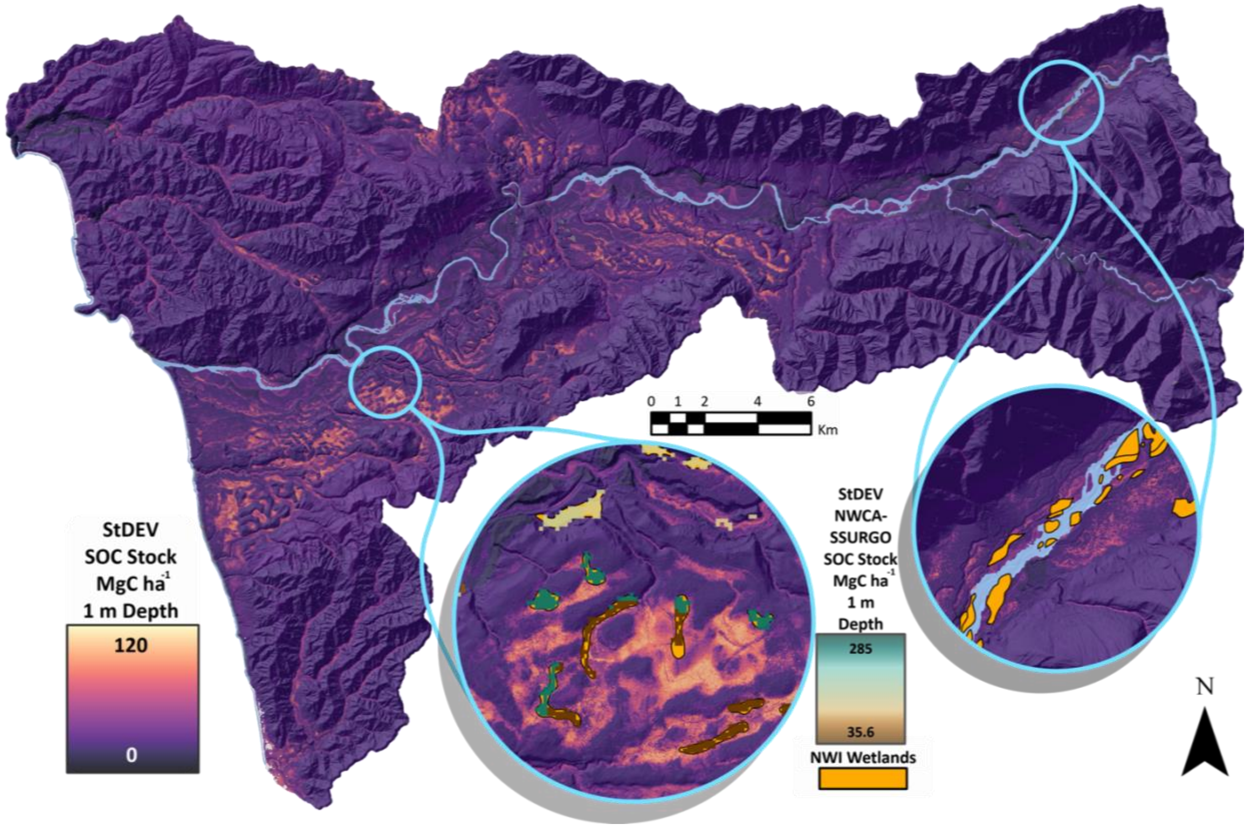


Supplemental Figure 4. **Standard deviation of modeled 1 m soil organic carbon (SOC) stocks from bootstrapping in the Hoh River Watershed (HRW).** Insets show both National Wetland Inventory (NWI) wetland locations and wetland SOC stocks from National Wetland Condition Assessment and Soil Survey Geographic Database dataset (NWCA-SSURGO) from Uhran et al. (2021)[3]. NWCA-SSURGO wetlands with SOC values are derived from the standard deviation in Uhran et al. (2021)[3]. We added a hill shade layer to highlight terrain and removed the river surface water shown in light blue for the final prediction map.

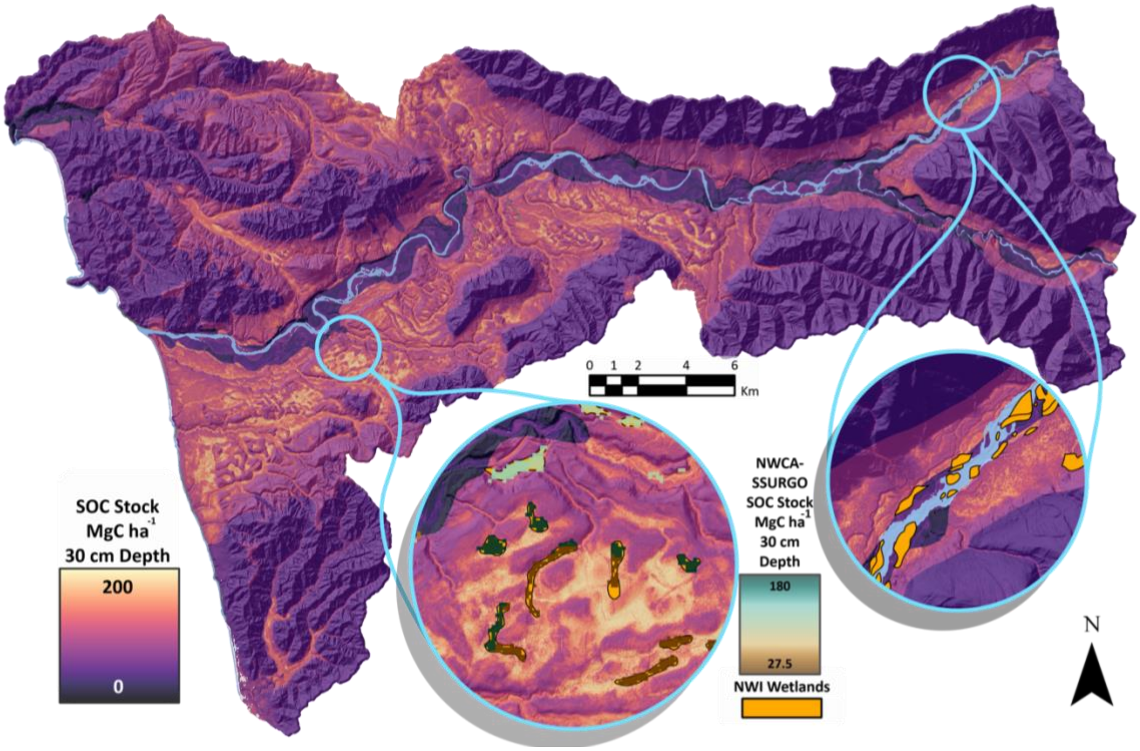


Supplemental Figure 5. **Predicted mean 30 cm soil organic carbon (SOC) stocks across the Hoh River Watershed (HRW.)** Insets show both National Wetland Inventory (NWI) wetland locations and SOC stocks from National Wetland Condition Assessment and Soil Survey Geographic Database dataset (NWCA-SSURGO) from Uhran et al. (2021)[3]. We added a hill shade layer to highlight terrain and removed the river surface water shown in light blue for the final prediction map.

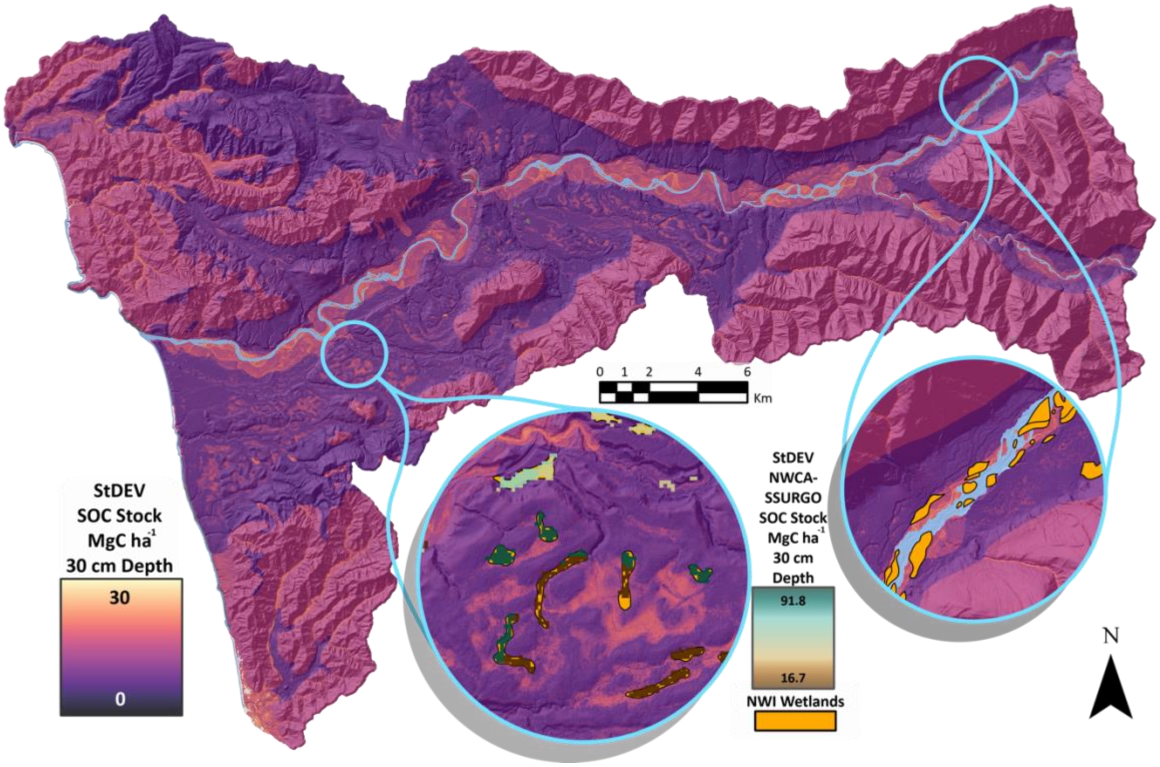


Supplemental Figure 6. **Standard deviation of modeled 30 cm soil organic carbon (SOC) stocks from bootstrapping in the Hoh River Watershed (HRW).** Insets show both National Wetland Inventory (NWI) wetland locations and wetland SOC stocks from National Wetland Condition Assessment and Soil Survey Geographic Database dataset (NWCA-SSURGO) from Uhran et al. (2021)[3]. NWCA-SSURGO wetlands with SOC values are derived from the standard deviation in Uhran et al. (2021)[3]. We added a hill shade layer to highlight terrain and removed the river surface water shown in light blue for the final prediction map.

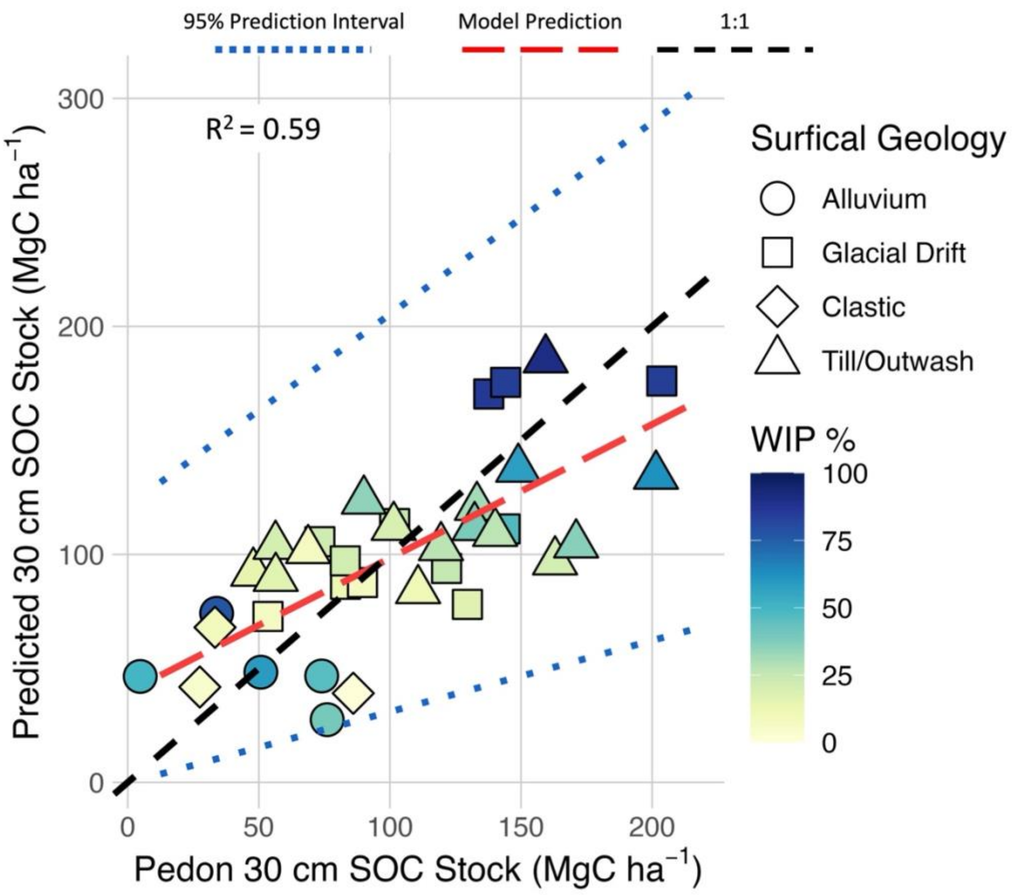


Supplementary Figure 7. **Model predicted soil organic carbon (SOC) stock compared to field sampled pedon SOC stock with a gradient of probability from the Wetland Intrinsic Potential (WIP) tool for the 30 cm depth.** Modeled SOC stocks were back transformed from square root values in the original linear mixed effects model with the fixed effect of WIP probability and random effect of surficial geology. Prediction intervals are based on bootstrapped 95% confidence intervals.

Supplementary Table 2. **Combined WIP and NWCA-SSURGO wetland SOC metrics for 1 m and 30 cm depth SOC stocks across the HRW.** The ± indicates the standard deviation and the values in parentheses indicate 95% confidence intervals of the bootstrapped model predictions for the WIP derived estimates and published standard deviations for the NWCA-SSURGO datasets in Uhran et al. (2021)[3]. Combined mean SOC stocks were calculated on a weighted area basis shown by the percentages. Forested wetlands were determined by masking maps of wetland SOC stocks canopy cover ≥ 50%

| | **Surface Area** | | | **30cm** | | **1m** | |
|---|---|---|---|---|---|---|---|
| **Landscape Class** | **Combined Surface (ha)** | **WIP (% of Total) (ha)** | **NWCA-SSURGO Surface Area (% of Total) (ha)** | **Area Weighted Mean SOC Stock (MgC ha$^{-1}$)** | **Sum Total SOC Stock (TgC)** | **Area Weighted Mean SOC Stock (MgC ha$^{-1}$)** | **Sum Total SOC Stock (TgC)** |
| Wetland | 6949 | 5308 (76%) | 1640 (24%) | 114 ± 22.9 | 0.8 ± 0.1 | 253 ± 63.1 | 1.8 ± 0.2 |
| Forested Wetland | 5678 | 4236 (75%) | 1442 (25%) | 117 ± 23.3 | 0.7 ± 0.1 | 264 ± 65.3 | 1.5 ± 0.2 |

Supplementary Table 3. **Wetland surface area size class distribution using individual wetland surface area quantiles.** The Size Classes group shows metrics for each individual percentile size class. The Cumulative Sum group shows the increasing sum over each quantile approaching 100% of all wetlands. Soil organic carbon (SOC) stock and Area within the Size Classes group were calculated as the sum within the wetland surface area size class for all wetlands above 0.0064 ha. Mean SOC stock was calculated as the mean SOC stock within each wetland and across all wetlands within the quantile size class.

| | **Size Classes** | | | | **Cumulative Sum** | | |
|---|---|---|---|---|---|---|---|
| **Quantiles of Wetland Area (ha)** | **SOC Stock (Tg)** | **Mean SOC Stock (Mg ha$^{-1}$)** | **Count** | **Area (ha)** | **SOC Stock (Tg)** | **Area (ha)** | **Count** |
| 0-5% (0.006 ha) | 0.0101 | 254 | 6216 | 40 | 0.0101 | 39.8 | 6216 |
| 5-25% (0.006-0.008) | 0.00878 | 252 | 4375 | 35 | 0.0189 | 74.5 | 10591 |
| 25-50% (0.008-0.011) | 0.0142 | 254 | 5467 | 56 | 0.0331 | 130 | 16058 |
| 50-75% (0.011-0.026) | 0.0348 | 254 | 7973 | 137 | 0.0678 | 267 | 24031 |
| 75-96.4% (0.026-0.40) | 0.143 | 252 | 6808 | 560 | 0.211 | 827 | 30839 |
| 96.4-100% (0.40-401) | 1.42 | 264 | 1142 | 5010 | 1.63 | 5838 | 31981 |

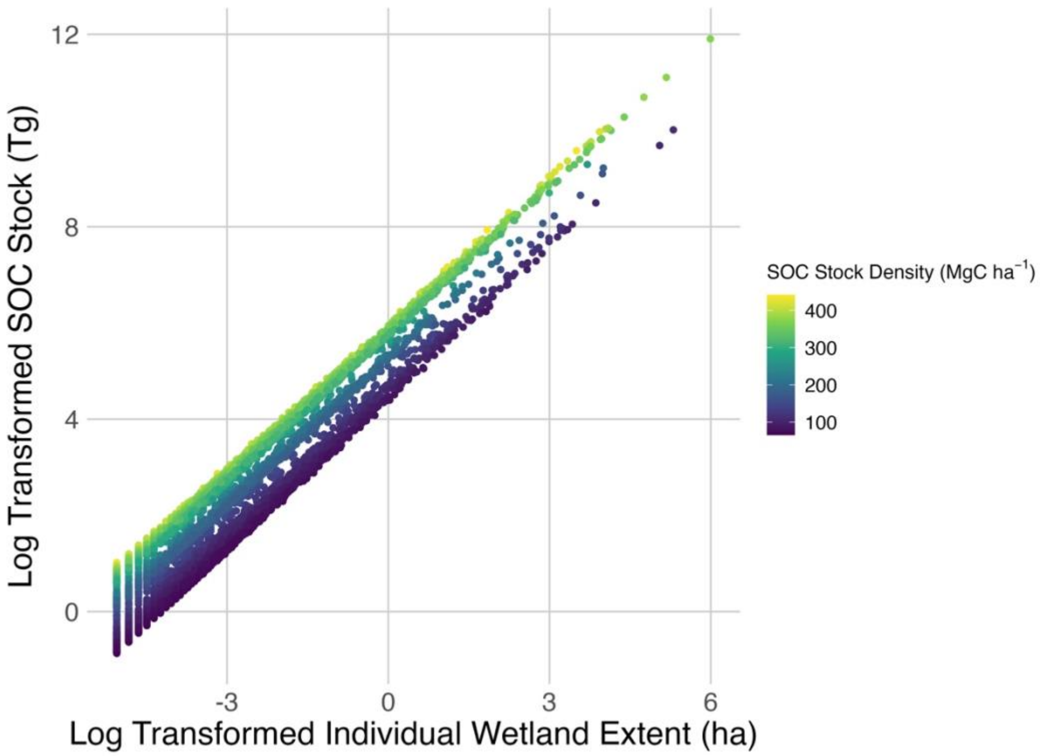


Supplemental Figure 8. **Log transformed extent for individual wetlands in relation to log transform soil organic carbon (SOC) stock for individual wetlands**. Points are colored by mean SOC stock density in each wetland.

Supplementary Table 4. **Comparison of total soil organic carbon (SOC) stock for the entire Hoh River Watershed HRW between the Wetland Intrinsic Potential (WIP) SOC model and global map products.** Global map products include the SoilGrids 2.0[4] model and the United Nations Food and Agriculture Organization (UN FAO) Global Soil Organic Carbon (GSOC) 1.5 map [5]. The ± indicates the standard deviation of the bootstrapped model predictions for the WIP derived estimates and published uncertainty ranges for SoilGrids and UN FAO GSOC. It should be noted that GSOC uncertainty maps contained incomplete data for the HRW, covering only 2996 ha. Therefore mean SOC stock uncertainty was extrapolated to the full HRW area for a total GSOC SOC stock uncertainty.

| | | 30cm Depth | |
|---|---|---|---|
| **Source** | **Surface Area (ha)** | **Mean SOC Stock (MgC ha$^{-1}$)** | **Total SOC Stock (TgC)** |
| WIP | 68,145 | 72.9 ± 12.5 | 5.0 ± 0.9 |
| SoilGrids 2.0 | 68,145 | 115 ± 15.8 | 7.8 ± 1.1 |
| UN FAO GSOC 1.5 | 68,145 | 56.9 ± 50.2 | 3.9 ± 3.4 |

Supplementary References:


1. Halabisky, M. *et al.* The wetland intrinsic potential tool: Mapping wetland intrinsic potential through machine learning of multi-scale remote sensing proxies of wetland indicators. *Hydrol. Earth Syst. Sci.* **27**, 3687–3699 (2023).
2. Maxwell, A. E., Warner, T. A. & Strager, M. P. Predicting palustrine wetland probability using random forest machine learning and digital elevation data-derived terrain variables. *Photogramm. Eng. Remote Sens.* **82**, 437–447 (2016).
3. Uhran, B. *et al.* Improved wetland soil organic carbon stocks of the conterminous U.S. through data harmonization. *Front. Soil Sci.* **1**, (2021).
4. Poggio, L. *et al.* SoilGrids 2.0: Producing soil information for the globe with quantified spatial uncertainty. *SOIL* **7**, 217–240 (2021).
5. FAO. *Global soil organic carbon map (GSOCmap): technical report*. (FAO, 2018).
